# Crystal structures of 4-{(*E*)-3-[(imino-λ^5^-aza­nyl­idene)amino]­prop-1-en­yl}-*N*,*N*-di­methyl­imidazole-1-sulfonamide and 2-[(imino-λ^5^-aza­nyl­idene)amino]-4-{(*E*)-3-[(imino-λ^5^-aza­nyl­idene)amino]­prop-1-en­yl}-*N*,*N*-di­methyl­imidazole-1-sulfonamide

**DOI:** 10.1107/S205698901900519X

**Published:** 2019-04-25

**Authors:** Lorenzo M. Cruz, Raakiyah Y. Moore, Marcela Torres Gutierrez, Apsara K. Herath, Carl J. Lovely, Muhammed Yousufuddin

**Affiliations:** a Life and Health Sciences Department, University of North Texas at Dallas, 7400 University Hills Blvd, Dallas, TX 75241, USA; b Department of Chemistry and Biochemistry, University of Texas at Arlington, 700 Planetarium Pl., Arlington, TX 76019, USA

**Keywords:** crystal structure, imidazole, nagelamide, azide, amide

## Abstract

The structures of two azide containing imidazole derivatives are reported. Allylic azides are fairly reactive making them attractive starting compounds to convert into amides. The first, C_8_H_12_N_6_O_2_S, contains one azide group with an N_α_—N_β_ distance of 1.229 (2) Å and an N_β_—N_γ_ distance of 1.128 (2) Å. The second, C_8_H_11_N_9_O_2_S, contains two azide groups with an average N_α_—N_β_ distance of 1.249 (2) Å and an average N_β_—N_γ_ distance of 1.132 (2) Å. Each compound contains a bulky protecting group (di­methyl­amino­sulfon­yl) which can be easily removed under mildly acidic conditions.

## Abstract 2

The structures of two azide containing imidazole derivatives are reported. The first, C_8_H_12_N_6_O_2_S, contains one azide group with an N_α_—N_β_ distance of 1.229 (2) Å and an N_β_—N_γ_ distance of 1.128 (2) Å. The second, C_8_H_11_N_9_O_2_S, contains two azide groups with an average N_α_—N_β_ distance of 1.249 (2) Å and an average N_β_—N_γ_ distance of 1.132 (2) Å. Each compound contains a bulky protecting group.

## Chemical context   

**Figure 1:**
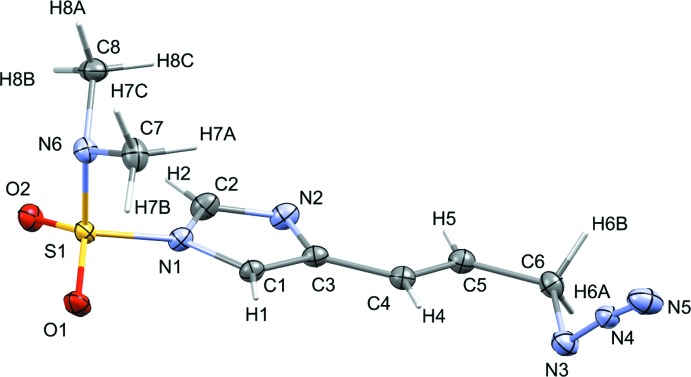
The mol­ecular structure of compound **1**, with atom labels and 50% probability displacement ellipsoids for non-H atoms.

The efficient synthesis of nagelamide alkaloids (a subfamily of oroidin natural products derived from marine sponges) has garnered inter­est (Du *et al.*, 2006 ▸; Das *et al.*, 2016 ▸) since first reported (Endo *et al.*, 2004 ▸). Allylic azides (Carlson & Top­czewski, 2019 ▸) are fairly reactive making them attractive starting compounds to convert into amides. Our group has successfully synthesized a number of azide-containing imidazole derivatives and determined their structures. Many of our strategies have led to the successful synthesis of several nagelamide derivatives (Bhandari *et al.*, 2009 ▸; Mukherjee *et al.*, 2010 ▸). However, the application of our approaches to several other nagelamide congeners were unsuccessful, leading us to rethink our tactics. Recently, we reported the efficient synthesis of amide compounds from allylic azide-containing imidazoles (Herath *et al.*, 2017 ▸). In that report we were also able to show that although the imidazoles contained di­methyl­amino­sulfonyl (DMAS) protecting groups, efficient conversion to amides was still possible. In addition, the free imidazole (lacking the protecting group but still containing azide) underwent selective and rapid conversion to amide without the undesired hydro­sulfenylation we observed with protected imidazoles. Here we present the crystal structures of two azide-containing imidazoles, 4-{(*E*)-3-[(imino-λ^5^-aza­nyl­idene)amino]­prop-1-en­yl}-*N*,*N*-di­methyl­imidazole-1-sulfonamide (**1**) and 2-[(imino-λ^5^-aza­nyl­idene)amino]-4-{(*E*)-3-[(imino-λ^5^-aza­nyl­idene)amino]­prop-1-en­yl}-*N*,*N*-di­methyl­imidazole-1-sulfonamide (**2**). These compounds were synthesized in the previous study but the structures were not reported. Figs. 1 ▸ and 2 ▸ show displacement ellipsoid plots of **1** and **2**, respectively.
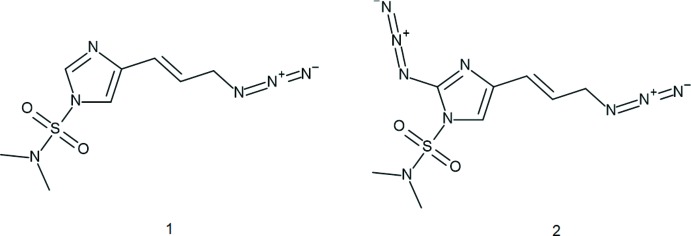


## Structural commentary   

Compound **1** contains one allylic azide while compound **2** contains two azide groups, an allylic azide and one azide bound directly to the imidazole ring at C2. The azide group in **1** shows an N3—N4 distance of 1.229 (2) Å and an N4—N5 distance of 1.128 (2) Å. The N3—N4—N5 angle is 172.32 (13)°. The azide groups in **2** show an N3—N4 distance of 1.253 (2) Å, N4—N5 distance of 1.129 (2) Å, N6—N7 distance of 1.239 (2) Å, and N7—N8 distance of 1.134 (2) Å. The N3—N4—N5 angle is 171.58 (15)° and the N6—N7—N8 angle is 173.95 (15)°. All three azide moieties in both compounds show the same general trend of a longer N_α_—N_β_ distance and shorter N_β_—N_γ_ distance with a quasilinear geometry. This is typical for covalent azides with terminal N_β_—N_γ_ demonstrating more triple-bond character. A previously reported covalent azide occurring in the compound ethyl-2-[(azido­carbon­yl)amino]­benzoate demonstrated bond lengths N_α_—N_β_ of 1.264 (2) Å and N_β_—N_γ_ of 1.131 (2) Å and an N_α_—N_β_—N_α_ angle of 174.7 (2)° (Yassine *et al.*, 2016 ▸).

**Figure 3:**
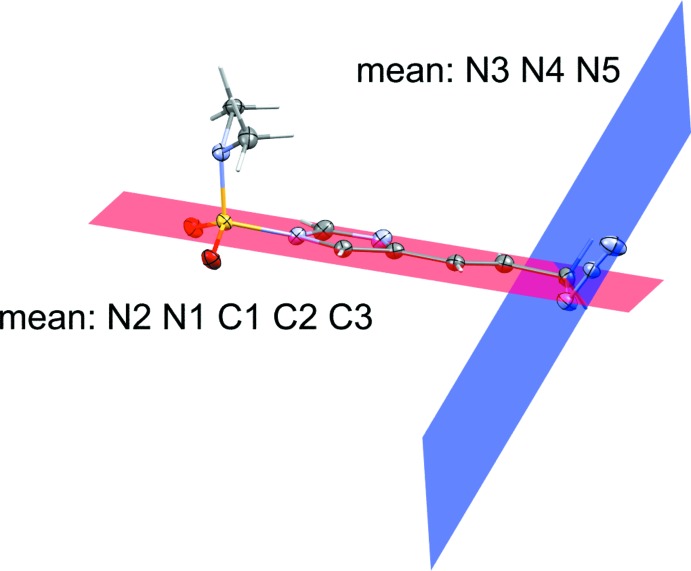
Dihedral planes between imidazole and allylic azide for compound **1**

The torsion angles for the azides and dihedral angles between the azides and imidazole rings for both compounds have been measured. The allylic azide torsion angles between **1** and **2** are quite different. The measured torsion angle for the allylic azide (C5—C6—N3—N4) in **1** was −115.21 (13)° while the related torsion angle (C5—C6—N6—N7) in **2** was 50.25 (18)°. **2** contains one azide group bound to the imidazole at C2 and shows a torsion angle N1—C2—N3—N4 of −174.82 (11)°. The allylic azides in both compounds exhibit a similar dihedral angle between the azide and the imidazole ring, 70.3 (11)° for **1** and 77.3 (17)° for **2**. While the imidazole-bound azide in **2** shows a dihedral angle of 5.0 (10)°. Indeed, the torsion angle and dihedral angle for this particular azide demonstrate the near planarity between the imidazole and its covalently bound azide. Figs. 3 ▸ and 4 ▸ show the dihedral planes for **1** and **2**, respectively.

Both title compounds contain a DMAS protecting group. The amine component of this protecting group is *sp* ^3^-hybrid­ized, as validated by the C—N—C bond angles C6—N6—C8 = 113.86 (10)° for **1** and C7—N9—C8 = 113.93 (12)° for **2**. Both compounds also contain a double bond between C4 and C5. The measured bond distance is 1.333 (2) Å for **1** and 1.340 (2) Å for **2**.

The imidazole ring in **1** is substituted at the N1 and C3 position with no substitution at C2. The N1—C2 distance is 1.378 (2) Å while the N2—C2 distance is 1.301 (2) Å. However, in **2**, the imidazole ring is substituted with an azide group at C2 but this seemingly has no effect on the ring bond distances. The measured bond distances for N1—C2 and N2—C2 in **2** are 1.385 (2) and 1.310 (2) Å, respectively.

There is, however, a significant difference in the measured N1—S1 distance for the two compounds. The imidazole ring is substituted at the N1 position for both compounds with DMAS. The N1—S1 distance for **1** is 1.686 (1) Å and 1.718 (1) Å for **2**. The disparity may be attributed to the presence of azide, which is substituted at the C2 position for **2**.

## Supra­molecular features   

**Figure 5:**
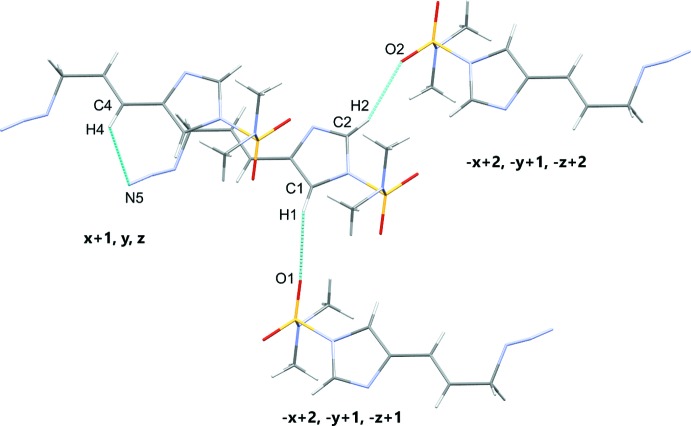
Close contacts for compound **1**.

The title compounds each contain bulky DMAS protecting groups and hydrogen bond distances that influence the mol­ecule packing. Compound **1** shows C1—H1⋯O1^i^ and C2—H2⋯O2^ii^ inter­actions of 2.53 and 2.39 Å, respectively. There is also a C4—H4⋯N5^iii^ inter­action of 2.70 Å (symmetry codes as in Table 1 ▸). Compound **2** demonstrates a C7—H7*B*⋯O1^i^ inter­action of 2.51 Å. There are also C6—H6*A*⋯N8^ii^ and C7—H7*C*⋯N6^iii^ inter­actions of 2.70 and 2.62 Å, respectively (symmetry codes as in Table 2 ▸). Figs. 5 ▸ and 6 ▸ show the close contacts for **1** and **2**, respectively.

Although both compounds contain aromatic rings, there appears to be no π-stacking present in the crystals of either compound. The stacking appears more staggered, most likely due to the presence of bulky DMAS groups on both compounds. However, the staggering in **1** appears more pronounced than in **2**. In other words, the mol­ecules are further apart in **1**. This is most likely due to the larger torsion angle for the azide in **1** than in **2**.

## Database survey   

A search of related compounds was conducted in the Cambridge Structural Database (Version 5.38; Groom *et al.*, 2016 ▸). One very closely related compound, methyl 3-(1-(di­methyl­sulfamo­yl)-1*H*-imidazol-5-yl)acrylate, was reported (Lovely *et al.*, 2010 ▸). This particular compound contains an imidazole with a DMAS protecting group and an allylic ester moiety. The DMAS amine has a C—N—C angle of 114.33 (14)°, showing the same amine hybridization exhibited in **1** and **2**. The C4=C5 double bond distance is measured to be 1.330 (2) Å which is similar to the bond distances in **1** and **2** [1.333 (2) and 1.340 (2) Å respectively].

The crystal structure of a related allylic azide has been reported from our previous study (Herath *et al.*, 2017 ▸). This particular compound is a dimerized mol­ecule with two allylic azides.

## Synthesis and crystallization   

**Figure 7:**
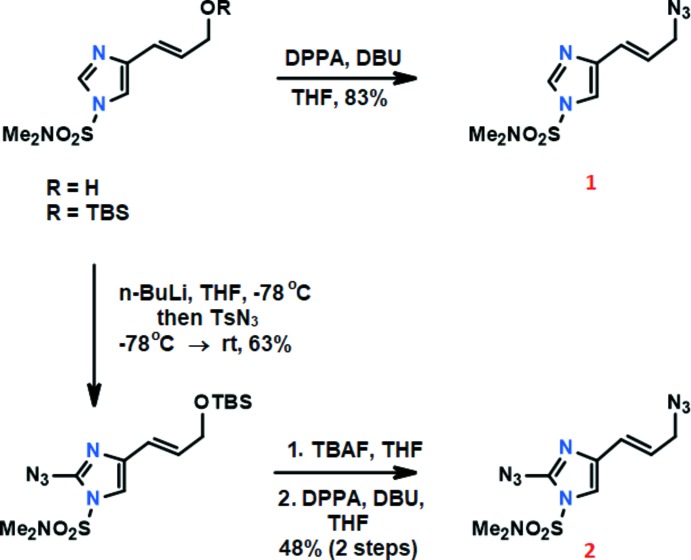
Synthetic scheme for both compounds. The title compounds are highlighted in red.

The syntheses of the title compounds were previously reported by our group (Lovely *et al.*, 2017 ▸). As shown in Fig. 7 ▸, the parent allylic azide **1** was prepared from the known alcohol starting compound (He *et al.*, 2003 ▸) by treatment with di­phenyl­phospho­rylazide (DPPA) and 1,8-di­aza­bicyclo­[5.4.0]undec-7-ene (DBU) according to the procedure described previously (Thompson *et al.*, 1993 ▸). Crystals were acquired by dissolving title compounds in ethanol with heating and slowly cooling in a freezer. Crystals appeared after about 1 week.

## Refinement   

Crystal data, data collection and structure refinement details for **1** and **2** are summarized in Table 3 ▸. Refinement for both compounds were routine. H atoms were positioned geometrically (C—H = 0.95–0.98 Å) and allowed to ride on their parent atoms, with *U*_iso_(H) = 1.5*U*_eq_(C) for methyl H and 1.2*U*_eq_(C) for other H atoms.

## Supplementary Material

Crystal structure: contains datablock(s) compound_1, compound_2. DOI: 10.1107/S205698901900519X/zl2753sup1.cif

Structure factors: contains datablock(s) compound_1. DOI: 10.1107/S205698901900519X/zl2753compound_1sup2.hkl

Structure factors: contains datablock(s) compound_2. DOI: 10.1107/S205698901900519X/zl2753compound_2sup3.hkl

Supporting information file. DOI: 10.1107/S205698901900519X/zl2753compound_1sup4.cml

Supporting information file. DOI: 10.1107/S205698901900519X/zl2753compound_2sup5.cml

CCDC references: 1910313, 1910312

Additional supporting information:  crystallographic information; 3D view; checkCIF report

## Figures and Tables

**Figure 2:**
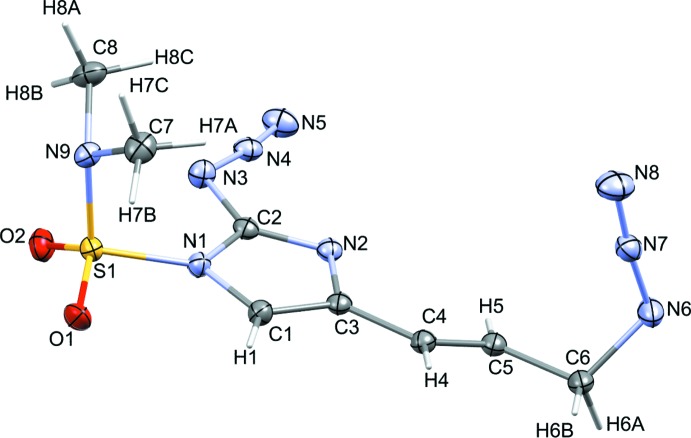
The mol­ecular structure of compound **2**, with atom labels and 50% probability displacement ellipsoids for non-H atoms.

**Figure 4:**
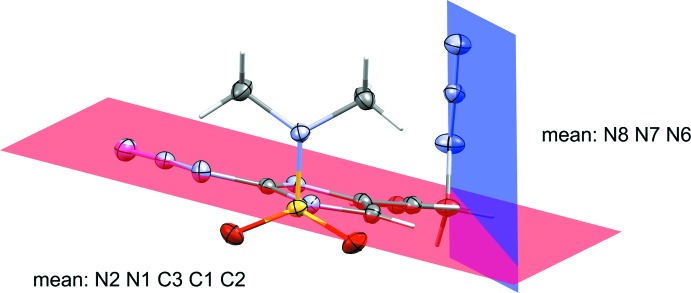
Dihedral planes between imidazole and allylic azide for compound **2**

**Figure 6:**
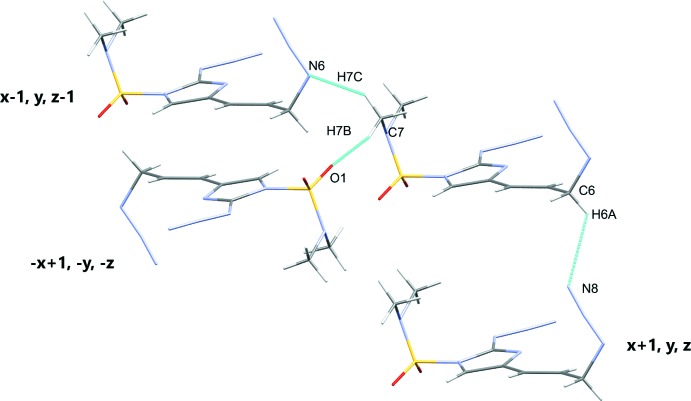
Close contacts for compound **2**.

**Table 1:** Hydrogen-bond geometry (Å, °) for **1** [Chem scheme1]

*D*—H⋯*A*	*D*—H	H⋯*A*	*D*⋯*A*	*D*—H⋯*A*
C1—H1⋯O1^i^	0.95	2.53	3.4487 (18)	164
C2—H2⋯O2^ii^	0.95	2.39	3.2861 (18)	157
C4—H4⋯N5^iii^	0.95	2.70	3.1920 (17)	113

Symmetry codes: (i) 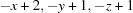; (ii) 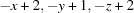; (iii) 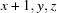.

**Table 2:** Hydrogen-bond geometry (Å, °) for **2** [Chem scheme1]

*D*—H⋯*A*	*D*—H	H⋯*A*	*D*⋯*A*	*D*—H⋯*A*
C7—H7*B*⋯O1^i^	0.98	2.51	3.444 (2)	160
C6—H6*A*⋯N8^ii^	0.99	2.70	3.337 (2)	123
C7—H7*C*⋯N6^iii^	0.98	2.62	3.357 (2)	132

Symmetry codes: (i) 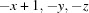; (ii) 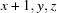; (iii) 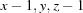.

**Table 3:** Experimental details

	**1**	**2**
Crystal data		
Chemical formula	C_8_H_12_N_6_O_2_S	C_8_H_11_N_9_O_2_S
*M*_r_	256.30	297.32
Crystal system, space group	Triclinic, *P* 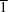	Triclinic, *P* 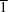
Temperature (K)	100	100
*a*, *b*, *c* (Å)	5.4252 (15), 9.830 (3), 11.137 (3)	6.6151 (18), 9.563 (3), 11.634 (3)
α, β, γ (°)	74.636 (5), 83.418 (5), 80.255 (5)	108.645 (4), 105.994 (4), 93.828 (4)
*V* (Å^3^)	563.0 (3)	660.6 (3)
*Z*	2	2
Radiation type	Mo *K*α	Mo *K*α
μ (mm^−1^)	0.29	0.26
Crystal size (mm)	0.25 × 0.20 × 0.05	0.80 × 0.28 × 0.08

Data collection		
Diffractometer	Bruker D8 Quest	Bruker D8 Quest
Absorption correction	Multi-scan (*SADABS*; Bruker, 2016 ▸)	Multi-scan (*SADABS*; Bruker, 2016 ▸)
*T*_min_, *T*_max_	0.616, 0.746	0.634, 0.747
No. of measured, independent and observed [*I* > 2σ(*I*)] reflections	9314, 4292, 3477	11030, 5171, 4051
*R*_int_	0.033	0.030
(sin θ/λ)_max_ (Å^−1^)	0.774	0.780

Refinement		
*R*[*F* ^2^ > 2σ(*F* ^2^)], *wR*(*F* ^2^), *S*	0.039, 0.104, 1.01	0.043, 0.109, 1.07
No. of reflections	4292	5171
No. of parameters	157	184
H-atom treatment	H-atom parameters constrained	H-atom parameters constrained
Δρ_max_, Δρ_min_ (e Å^−3^)	0.42, −0.48	0.46, −0.60

Computer programs: *APEX3* and *SAINT* (Bruker, 2016 ▸), *SHELXT2014* (Sheldrick, 2015*a* ▸), *SHELXL2017* (Sheldrick, 2015*b* ▸), *Mercury* (Macrae *et al.*, 2008 ▸) and *SHELXTL* (Sheldrick, 2008 ▸).

## supplementary crystallographic information

### 4-{(E)-3-[(Imino-λ5-azanylidene)amino]prop-1-enyl}-N,N-dimethylimidazole-1-sulfonamide (compound_1) Crystal data

**Table:**

C_8_H_12_N_6_O_2_S	*Z* = 2
*M**_r_* = 256.30	*F*(000) = 268
Triclinic, *P*1	*D*_x_ = 1.512 Mg m^−^^3^
*a* = 5.4252 (15) Å	Mo *K*α radiation, λ = 0.71073 Å
*b* = 9.830 (3) Å	Cell parameters from 4940 reflections
*c* = 11.137 (3) Å	θ = 3.2–33.3°
α = 74.636 (5)°	µ = 0.29 mm^−^^1^
β = 83.418 (5)°	*T* = 100 K
γ = 80.255 (5)°	Prism, colourless
*V* = 563.0 (3) Å^3^	0.25 × 0.20 × 0.05 mm

### 4-{(E)-3-[(Imino-λ5-azanylidene)amino]prop-1-enyl}-N,N-dimethylimidazole-1-sulfonamide (compound_1) Data collection

**Table:**

Bruker D8 Quest diffractometer	3477 reflections with *I* > 2σ(*I*)
φ and ω scans	*R*_int_ = 0.033
Absorption correction: multi-scan (SADABS; Bruker, 2016)	θ_max_ = 33.4°, θ_min_ = 3.2°
*T*_min_ = 0.616, *T*_max_ = 0.746	*h* = −8→8
9314 measured reflections	*k* = −15→15
4292 independent reflections	*l* = −17→17

### 4-{(E)-3-[(Imino-λ5-azanylidene)amino]prop-1-enyl}-N,N-dimethylimidazole-1-sulfonamide (compound_1) Refinement

**Table:**

Refinement on *F*^2^	Hydrogen site location: inferred from neighbouring sites
Least-squares matrix: full	H-atom parameters constrained
*R*[*F*^2^ > 2σ(*F*^2^)] = 0.039	*w* = 1/[σ^2^(*F*_o_^2^) + (0.0486*P*)^2^ + 0.2224*P*] where *P* = (*F*_o_^2^ + 2*F*_c_^2^)/3
*wR*(*F*^2^) = 0.104	(Δ/σ)_max_ = 0.001
*S* = 1.01	Δρ_max_ = 0.42 e Å^−^^3^
4292 reflections	Δρ_min_ = −0.48 e Å^−^^3^
157 parameters	Extinction correction: SHELXL2017 (Sheldrick, 2015b), Fc^*^=kFc[1+0.001xFc^2^λ^3^/sin(2θ)]^-1/4^
0 restraints	Extinction coefficient: 0.035 (6)
Primary atom site location: structure-invariant direct methods	

### 4-{(E)-3-[(Imino-λ5-azanylidene)amino]prop-1-enyl}-N,N-dimethylimidazole-1-sulfonamide (compound_1) Special details

**Table:**

Geometry. All esds (except the esd in the dihedral angle between two l.s. planes) are estimated using the full covariance matrix. The cell esds are taken into account individually in the estimation of esds in distances, angles and torsion angles; correlations between esds in cell parameters are only used when they are defined by crystal symmetry. An approximate (isotropic) treatment of cell esds is used for estimating esds involving l.s. planes.

### 4-{(E)-3-[(Imino-λ5-azanylidene)amino]prop-1-enyl}-N,N-dimethylimidazole-1-sulfonamide (compound_1) Fractional atomic coordinates and isotropic or equivalent isotropic displacement parameters (Å^2^)

**Table:**

	*x*	*y*	*z*	*U*_iso_*/*U*_eq_	
S1	1.02066 (5)	0.56887 (3)	0.72049 (3)	0.01267 (8)	
O1	1.14077 (17)	0.54225 (11)	0.60703 (8)	0.01878 (19)	
O2	1.15939 (17)	0.56915 (10)	0.82106 (8)	0.01797 (18)	
N1	0.84664 (19)	0.43637 (11)	0.77979 (9)	0.01388 (19)	
N2	0.6248 (2)	0.28893 (12)	0.91880 (10)	0.0177 (2)	
N3	0.1658 (2)	−0.00361 (13)	0.71066 (11)	0.0199 (2)	
N4	−0.0161 (2)	0.04337 (11)	0.64781 (10)	0.0164 (2)	
N5	−0.1668 (2)	0.08138 (15)	0.57975 (11)	0.0256 (3)	
N6	0.82889 (19)	0.71510 (11)	0.68919 (10)	0.01508 (19)	
C1	0.7216 (2)	0.37309 (13)	0.71178 (11)	0.0140 (2)	
H1	0.728564	0.389191	0.623584	0.017*	
C2	0.7775 (3)	0.38185 (14)	0.90406 (11)	0.0176 (2)	
H2	0.834333	0.408815	0.970797	0.021*	
C3	0.5864 (2)	0.28275 (13)	0.79888 (11)	0.0139 (2)	
C4	0.4234 (2)	0.19129 (12)	0.77555 (11)	0.0144 (2)	
H4	0.407866	0.192306	0.691209	0.017*	
C5	0.2939 (2)	0.10591 (13)	0.86438 (11)	0.0160 (2)	
H5	0.306699	0.106802	0.948514	0.019*	
C6	0.1298 (2)	0.00877 (14)	0.84143 (12)	0.0174 (2)	
H6A	0.168435	−0.086933	0.898507	0.021*	
H6B	−0.047919	0.045775	0.860031	0.021*	
C7	0.6774 (3)	0.73822 (15)	0.58222 (13)	0.0211 (3)	
H7A	0.536984	0.683286	0.607359	0.032*	
H7B	0.781837	0.706956	0.513923	0.032*	
H7C	0.612380	0.839831	0.554136	0.032*	
C8	0.6881 (3)	0.75732 (15)	0.79801 (13)	0.0211 (3)	
H8A	0.611434	0.857329	0.772839	0.032*	
H8B	0.802632	0.745666	0.863140	0.032*	
H8C	0.556684	0.697062	0.830419	0.032*	

### 4-{(E)-3-[(Imino-λ5-azanylidene)amino]prop-1-enyl}-N,N-dimethylimidazole-1-sulfonamide (compound_1) Atomic displacement parameters (Å^2^)

**Table:**

	*U*^11^	*U*^22^	*U*^33^	*U*^12^	*U*^13^	*U*^23^
S1	0.01100 (13)	0.01655 (14)	0.01073 (13)	−0.00187 (9)	−0.00111 (9)	−0.00383 (10)
O1	0.0157 (4)	0.0266 (5)	0.0153 (4)	−0.0035 (3)	0.0026 (3)	−0.0087 (4)
O2	0.0151 (4)	0.0247 (5)	0.0155 (4)	−0.0014 (3)	−0.0055 (3)	−0.0066 (3)
N1	0.0176 (5)	0.0138 (4)	0.0102 (4)	−0.0028 (4)	−0.0014 (3)	−0.0025 (3)
N2	0.0248 (5)	0.0166 (5)	0.0110 (4)	−0.0035 (4)	−0.0011 (4)	−0.0023 (4)
N3	0.0150 (5)	0.0256 (6)	0.0204 (5)	−0.0007 (4)	−0.0020 (4)	−0.0091 (4)
N4	0.0163 (5)	0.0170 (5)	0.0164 (5)	−0.0033 (4)	0.0022 (4)	−0.0057 (4)
N5	0.0231 (6)	0.0337 (7)	0.0202 (5)	0.0032 (5)	−0.0023 (4)	−0.0113 (5)
N6	0.0145 (4)	0.0154 (5)	0.0145 (4)	−0.0021 (4)	−0.0026 (4)	−0.0017 (4)
C1	0.0164 (5)	0.0154 (5)	0.0107 (5)	−0.0019 (4)	−0.0014 (4)	−0.0040 (4)
C2	0.0251 (6)	0.0176 (5)	0.0104 (5)	−0.0040 (5)	−0.0021 (4)	−0.0028 (4)
C3	0.0165 (5)	0.0133 (5)	0.0112 (5)	−0.0003 (4)	−0.0009 (4)	−0.0029 (4)
C4	0.0151 (5)	0.0145 (5)	0.0127 (5)	−0.0005 (4)	−0.0012 (4)	−0.0027 (4)
C5	0.0171 (5)	0.0174 (5)	0.0129 (5)	−0.0014 (4)	−0.0012 (4)	−0.0032 (4)
C6	0.0166 (5)	0.0190 (5)	0.0154 (5)	−0.0035 (4)	−0.0003 (4)	−0.0020 (4)
C7	0.0201 (6)	0.0213 (6)	0.0200 (6)	−0.0045 (5)	−0.0075 (5)	0.0014 (5)
C8	0.0207 (6)	0.0191 (6)	0.0231 (6)	0.0008 (5)	0.0013 (5)	−0.0079 (5)

### 4-{(E)-3-[(Imino-λ5-azanylidene)amino]prop-1-enyl}-N,N-dimethylimidazole-1-sulfonamide (compound_1) Geometric parameters (Å, º)

**Table:**

S1—O2	1.4200 (9)	C2—H2	0.9500
S1—O1	1.4209 (10)	C3—C4	1.4505 (17)
S1—N6	1.6067 (11)	C4—C5	1.3332 (17)
S1—N1	1.6858 (11)	C4—H4	0.9500
N1—C2	1.3783 (15)	C5—C6	1.4963 (18)
N1—C1	1.3896 (15)	C5—H5	0.9500
N2—C2	1.3012 (17)	C6—H6A	0.9900
N2—C3	1.3934 (15)	C6—H6B	0.9900
N3—N4	1.2288 (15)	C7—H7A	0.9800
N3—C6	1.4814 (17)	C7—H7B	0.9800
N4—N5	1.1277 (16)	C7—H7C	0.9800
N6—C7	1.4711 (16)	C8—H8A	0.9800
N6—C8	1.4736 (17)	C8—H8B	0.9800
C1—C3	1.3668 (16)	C8—H8C	0.9800
C1—H1	0.9500		
			
O2—S1—O1	121.78 (6)	C5—C4—C3	124.48 (11)
O2—S1—N6	108.51 (6)	C5—C4—H4	117.8
O1—S1—N6	108.95 (6)	C3—C4—H4	117.8
O2—S1—N1	104.30 (6)	C4—C5—C6	124.87 (11)
O1—S1—N1	105.35 (5)	C4—C5—H5	117.6
N6—S1—N1	106.96 (6)	C6—C5—H5	117.6
C2—N1—C1	106.84 (10)	N3—C6—C5	111.73 (10)
C2—N1—S1	126.59 (9)	N3—C6—H6A	109.3
C1—N1—S1	126.18 (8)	C5—C6—H6A	109.3
C2—N2—C3	105.77 (10)	N3—C6—H6B	109.3
N4—N3—C6	116.05 (11)	C5—C6—H6B	109.3
N5—N4—N3	172.32 (13)	H6A—C6—H6B	107.9
C7—N6—C8	113.86 (10)	N6—C7—H7A	109.5
C7—N6—S1	116.58 (9)	N6—C7—H7B	109.5
C8—N6—S1	115.49 (9)	H7A—C7—H7B	109.5
C3—C1—N1	105.29 (10)	N6—C7—H7C	109.5
C3—C1—H1	127.4	H7A—C7—H7C	109.5
N1—C1—H1	127.4	H7B—C7—H7C	109.5
N2—C2—N1	111.73 (11)	N6—C8—H8A	109.5
N2—C2—H2	124.1	N6—C8—H8B	109.5
N1—C2—H2	124.1	H8A—C8—H8B	109.5
C1—C3—N2	110.36 (11)	N6—C8—H8C	109.5
C1—C3—C4	127.00 (11)	H8A—C8—H8C	109.5
N2—C3—C4	122.65 (11)	H8B—C8—H8C	109.5

### 4-{(E)-3-[(Imino-λ5-azanylidene)amino]prop-1-enyl}-N,N-dimethylimidazole-1-sulfonamide (compound_1) Hydrogen-bond geometry (Å, º)

**Table:**

*D*—H···*A*	*D*—H	H···*A*	*D*···*A*	*D*—H···*A*
C1—H1···O1^i^	0.95	2.53	3.4487 (18)	164
C2—H2···O2^ii^	0.95	2.39	3.2861 (18)	157
C4—H4···N5^iii^	0.95	2.70	3.1920 (17)	113

Symmetry codes: (i) −*x*+2, −*y*+1, −*z*+1; (ii) −*x*+2, −*y*+1, −*z*+2; (iii) *x*+1, *y*, *z*.

### 2-[(Imino-λ5-azanylidene)amino]-4-{(E)-3-[(imino-λ5-azanylidene)amino]prop-1-enyl}-N,N-dimethylimidazole-1-sulfonamide (compound_2) Crystal data

**Table:**

C_8_H_11_N_9_O_2_S	*Z* = 2
*M**_r_* = 297.32	*F*(000) = 308
Triclinic, *P*1	*D*_x_ = 1.495 Mg m^−^^3^
*a* = 6.6151 (18) Å	Mo *K*α radiation, λ = 0.71073 Å
*b* = 9.563 (3) Å	Cell parameters from 5257 reflections
*c* = 11.634 (3) Å	θ = 3.2–33.6°
α = 108.645 (4)°	µ = 0.26 mm^−^^1^
β = 105.994 (4)°	*T* = 100 K
γ = 93.828 (4)°	Needle, colourless
*V* = 660.6 (3) Å^3^	0.80 × 0.28 × 0.08 mm

### 2-[(Imino-λ5-azanylidene)amino]-4-{(E)-3-[(imino-λ5-azanylidene)amino]prop-1-enyl}-N,N-dimethylimidazole-1-sulfonamide (compound_2) Data collection

**Table:**

Bruker D8 Quest diffractometer	4051 reflections with *I* > 2σ(*I*)
φ and ω scans	*R*_int_ = 0.030
Absorption correction: multi-scan (SADABS; Bruker, 2016)	θ_max_ = 33.7°, θ_min_ = 3.2°
*T*_min_ = 0.634, *T*_max_ = 0.747	*h* = −10→10
11030 measured reflections	*k* = −14→14
5171 independent reflections	*l* = −17→18

### 2-[(Imino-λ5-azanylidene)amino]-4-{(E)-3-[(imino-λ5-azanylidene)amino]prop-1-enyl}-N,N-dimethylimidazole-1-sulfonamide (compound_2) Refinement

**Table:**

Refinement on *F*^2^	Hydrogen site location: inferred from neighbouring sites
Least-squares matrix: full	H-atom parameters constrained
*R*[*F*^2^ > 2σ(*F*^2^)] = 0.043	*w* = 1/[σ^2^(*F*_o_^2^) + (0.0401*P*)^2^ + 0.3565*P*] where *P* = (*F*_o_^2^ + 2*F*_c_^2^)/3
*wR*(*F*^2^) = 0.109	(Δ/σ)_max_ < 0.001
*S* = 1.07	Δρ_max_ = 0.46 e Å^−^^3^
5171 reflections	Δρ_min_ = −0.60 e Å^−^^3^
184 parameters	Extinction correction: SHELXL2017 (Sheldrick, 2015b), Fc^*^=kFc[1+0.001xFc^2^λ^3^/sin(2θ)]^-1/4^
0 restraints	Extinction coefficient: 0.022 (3)
Primary atom site location: structure-invariant direct methods	

### 2-[(Imino-λ5-azanylidene)amino]-4-{(E)-3-[(imino-λ5-azanylidene)amino]prop-1-enyl}-N,N-dimethylimidazole-1-sulfonamide (compound_2) Special details

**Table:**

Geometry. All esds (except the esd in the dihedral angle between two l.s. planes) are estimated using the full covariance matrix. The cell esds are taken into account individually in the estimation of esds in distances, angles and torsion angles; correlations between esds in cell parameters are only used when they are defined by crystal symmetry. An approximate (isotropic) treatment of cell esds is used for estimating esds involving l.s. planes.

### 2-[(Imino-λ5-azanylidene)amino]-4-{(E)-3-[(imino-λ5-azanylidene)amino]prop-1-enyl}-N,N-dimethylimidazole-1-sulfonamide (compound_2) Fractional atomic coordinates and isotropic or equivalent isotropic displacement parameters (Å^2^)

**Table:**

	*x*	*y*	*z*	*U*_iso_*/*U*_eq_	
S1	0.58817 (5)	0.32361 (4)	0.11881 (3)	0.01478 (8)	
O1	0.65163 (17)	0.18829 (12)	0.05384 (10)	0.0219 (2)	
O2	0.66446 (17)	0.46415 (12)	0.11471 (10)	0.0215 (2)	
N1	0.68124 (18)	0.33810 (13)	0.27604 (10)	0.0146 (2)	
N2	0.75962 (17)	0.42326 (13)	0.48977 (10)	0.0144 (2)	
N3	0.64496 (19)	0.59233 (13)	0.37520 (11)	0.0174 (2)	
N4	0.67946 (18)	0.69635 (13)	0.48007 (11)	0.0164 (2)	
N5	0.7040 (2)	0.80092 (15)	0.56596 (13)	0.0242 (3)	
N6	0.8624 (2)	0.18310 (17)	0.83285 (12)	0.0237 (3)	
N7	0.66897 (19)	0.15149 (14)	0.77329 (12)	0.0193 (2)	
N8	0.4889 (2)	0.12679 (17)	0.72893 (15)	0.0287 (3)	
N9	0.33102 (18)	0.29742 (13)	0.07962 (11)	0.0156 (2)	
C1	0.7358 (2)	0.21797 (15)	0.31603 (13)	0.0156 (2)	
H1	0.738643	0.119381	0.263572	0.019*	
C2	0.69706 (19)	0.45659 (15)	0.38610 (12)	0.0139 (2)	
C3	0.78417 (19)	0.27227 (15)	0.44638 (12)	0.0144 (2)	
C4	0.8525 (2)	0.19077 (15)	0.53300 (12)	0.0156 (2)	
H4	0.836577	0.084831	0.497346	0.019*	
C5	0.9364 (2)	0.25679 (16)	0.66005 (13)	0.0159 (2)	
H5	0.954749	0.362879	0.695491	0.019*	
C6	1.0028 (2)	0.17314 (17)	0.74931 (13)	0.0181 (3)	
H6A	1.152396	0.214636	0.803867	0.022*	
H6B	0.996255	0.066642	0.699097	0.022*	
C7	0.2306 (2)	0.15853 (17)	0.08670 (15)	0.0221 (3)	
H7A	0.250179	0.171110	0.176261	0.033*	
H7B	0.297367	0.074636	0.048982	0.033*	
H7C	0.077775	0.137969	0.039540	0.033*	
C8	0.2288 (2)	0.42873 (17)	0.12339 (15)	0.0221 (3)	
H8A	0.079649	0.408488	0.069371	0.033*	
H8B	0.304630	0.516510	0.117531	0.033*	
H8C	0.234224	0.448176	0.212252	0.033*	

### 2-[(Imino-λ5-azanylidene)amino]-4-{(E)-3-[(imino-λ5-azanylidene)amino]prop-1-enyl}-N,N-dimethylimidazole-1-sulfonamide (compound_2) Atomic displacement parameters (Å^2^)

**Table:**

	*U*^11^	*U*^22^	*U*^33^	*U*^12^	*U*^13^	*U*^23^
S1	0.01499 (14)	0.01799 (16)	0.01287 (14)	0.00388 (11)	0.00554 (11)	0.00623 (11)
O1	0.0251 (5)	0.0260 (5)	0.0168 (5)	0.0108 (4)	0.0107 (4)	0.0057 (4)
O2	0.0222 (5)	0.0242 (5)	0.0211 (5)	−0.0001 (4)	0.0076 (4)	0.0122 (4)
N1	0.0157 (5)	0.0157 (5)	0.0123 (4)	0.0037 (4)	0.0040 (4)	0.0049 (4)
N2	0.0120 (4)	0.0159 (5)	0.0151 (5)	0.0010 (4)	0.0043 (4)	0.0055 (4)
N3	0.0198 (5)	0.0148 (5)	0.0169 (5)	0.0027 (4)	0.0053 (4)	0.0052 (4)
N4	0.0130 (5)	0.0164 (5)	0.0206 (5)	0.0021 (4)	0.0064 (4)	0.0066 (4)
N5	0.0221 (6)	0.0208 (6)	0.0261 (6)	0.0026 (5)	0.0090 (5)	0.0025 (5)
N6	0.0176 (5)	0.0379 (7)	0.0179 (5)	0.0039 (5)	0.0057 (4)	0.0129 (5)
N7	0.0188 (5)	0.0212 (6)	0.0225 (6)	0.0048 (4)	0.0100 (4)	0.0105 (5)
N8	0.0185 (6)	0.0307 (7)	0.0381 (8)	0.0026 (5)	0.0097 (5)	0.0134 (6)
N9	0.0139 (5)	0.0152 (5)	0.0169 (5)	0.0026 (4)	0.0030 (4)	0.0060 (4)
C1	0.0154 (5)	0.0160 (6)	0.0160 (5)	0.0034 (4)	0.0047 (4)	0.0063 (5)
C2	0.0106 (5)	0.0148 (5)	0.0159 (5)	0.0004 (4)	0.0044 (4)	0.0051 (4)
C3	0.0107 (5)	0.0169 (6)	0.0156 (5)	0.0015 (4)	0.0037 (4)	0.0063 (5)
C4	0.0131 (5)	0.0178 (6)	0.0168 (6)	0.0032 (4)	0.0044 (4)	0.0076 (5)
C5	0.0132 (5)	0.0193 (6)	0.0167 (6)	0.0031 (4)	0.0053 (4)	0.0078 (5)
C6	0.0141 (5)	0.0254 (7)	0.0167 (6)	0.0050 (5)	0.0051 (4)	0.0093 (5)
C7	0.0192 (6)	0.0195 (7)	0.0245 (7)	−0.0017 (5)	0.0028 (5)	0.0080 (5)
C8	0.0180 (6)	0.0203 (7)	0.0251 (7)	0.0074 (5)	0.0042 (5)	0.0057 (5)

### 2-[(Imino-λ5-azanylidene)amino]-4-{(E)-3-[(imino-λ5-azanylidene)amino]prop-1-enyl}-N,N-dimethylimidazole-1-sulfonamide (compound_2) Geometric parameters (Å, º)

**Table:**

S1—O2	1.4237 (11)	C1—C3	1.3707 (18)
S1—O1	1.4297 (11)	C1—H1	0.9500
S1—N9	1.6146 (12)	C3—C4	1.4579 (18)
S1—N1	1.7177 (12)	C4—C5	1.3402 (19)
N1—C2	1.3846 (17)	C4—H4	0.9500
N1—C1	1.4039 (17)	C5—C6	1.4961 (19)
N2—C2	1.3099 (17)	C5—H5	0.9500
N2—C3	1.4058 (18)	C6—H6A	0.9900
N3—N4	1.2531 (16)	C6—H6B	0.9900
N3—C2	1.4002 (18)	C7—H7A	0.9800
N4—N5	1.1291 (17)	C7—H7B	0.9800
N6—N7	1.2389 (17)	C7—H7C	0.9800
N6—C6	1.5053 (18)	C8—H8A	0.9800
N7—N8	1.1342 (18)	C8—H8B	0.9800
N9—C8	1.4792 (18)	C8—H8C	0.9800
N9—C7	1.4814 (19)		
			
O2—S1—O1	121.70 (7)	N2—C3—C4	122.43 (12)
O2—S1—N9	109.81 (6)	C5—C4—C3	123.73 (13)
O1—S1—N9	108.59 (6)	C5—C4—H4	118.1
O2—S1—N1	106.16 (6)	C3—C4—H4	118.1
O1—S1—N1	102.88 (6)	C4—C5—C6	123.82 (13)
N9—S1—N1	106.52 (6)	C4—C5—H5	118.1
C2—N1—C1	105.71 (11)	C6—C5—H5	118.1
C2—N1—S1	129.94 (10)	C5—C6—N6	111.84 (11)
C1—N1—S1	124.00 (9)	C5—C6—H6A	109.2
C2—N2—C3	104.81 (11)	N6—C6—H6A	109.2
N4—N3—C2	114.19 (12)	C5—C6—H6B	109.2
N5—N4—N3	171.58 (15)	N6—C6—H6B	109.2
N7—N6—C6	113.91 (12)	H6A—C6—H6B	107.9
N8—N7—N6	173.95 (15)	N9—C7—H7A	109.5
C8—N9—C7	113.93 (12)	N9—C7—H7B	109.5
C8—N9—S1	117.77 (10)	H7A—C7—H7B	109.5
C7—N9—S1	115.55 (9)	N9—C7—H7C	109.5
C3—C1—N1	105.82 (11)	H7A—C7—H7C	109.5
C3—C1—H1	127.1	H7B—C7—H7C	109.5
N1—C1—H1	127.1	N9—C8—H8A	109.5
N2—C2—N1	113.05 (12)	N9—C8—H8B	109.5
N2—C2—N3	128.30 (12)	H8A—C8—H8B	109.5
N1—C2—N3	118.64 (11)	N9—C8—H8C	109.5
C1—C3—N2	110.59 (11)	H8A—C8—H8C	109.5
C1—C3—C4	126.98 (13)	H8B—C8—H8C	109.5

### 2-[(Imino-λ5-azanylidene)amino]-4-{(E)-3-[(imino-λ5-azanylidene)amino]prop-1-enyl}-N,N-dimethylimidazole-1-sulfonamide (compound_2) Hydrogen-bond geometry (Å, º)

**Table:**

*D*—H···*A*	*D*—H	H···*A*	*D*···*A*	*D*—H···*A*
C7—H7*B*···O1^i^	0.98	2.51	3.444 (2)	160
C6—H6*A*···N8^ii^	0.99	2.70	3.337 (2)	123
C7—H7*C*···N6^iii^	0.98	2.62	3.357 (2)	132

Symmetry codes: (i) −*x*+1, −*y*, −*z*; (ii) *x*+1, *y*, *z*; (iii) *x*−1, *y*, *z*−1.
